# Notes and News

**Published:** 1900-05-12

**Authors:** 


### Notes and News.

HOSPITAL MEETINGS.

Victoria Hospital for Children, Chelsea.—Annual Meeting at four o'clock p.m., on Monday, May 14th.

Hampstead Hospital.—Festival Dinner at half-past seven o'clock p.m., on Wednesday, May 16th, at the Grand Hotel, W.C.

A case of plague is reported in a suburb of Brisbane.

Dr. John Wyllie has been appointed Professor of Medicine at Edinburgh, in place of the late Sir Thomas Grainger Stewart.

Sir Henry Harben will open the new "open-air" wing, which has been constructed to accommodate 40 patients, at the North London Hospital for Consumption, Hampstead, on June 20th.

The following lectures will take place at the Hospital for Sick Children, Great Ormond Street, in May: Thursday, May 17th, Mr. Thomas H. Kellock will give a Demonstration of Selected Cases; Thursday, May 24th, Mr. Bernard Pitts will give a Demonstration of Selected Cases; Thursday, May 31st, Dr. Still will lecture on "Meningitis in Children." All these lectures are free to medical practitioners, and will take place at four o'clock.

People are much accustomed to associate green dyes with arsenic and other deleterious materials, and the *Lancet*, being struck with the green colour of the new halfpenny stamp, has had it analysed, lest perchance in licking it we should take poison unawares. It is satisfactory to know, then, that the green tint of the new stamp is due to nothing worse than Prussian blue and a chrome colour, and that, so far as mineral ingredients are concerned, we may continue the convenient although æsthetically doubtful practice of licking stamps. The *Lancet*, however, hedges somewhat in regard to the gum. Chemically, it may be all right, but, as it says, "the bacteriology of the stamp has to be reckoned with," and it adds that there is little doubt that postage stamps are liable to be infected with pathogenic organisms. Would that we had nothing worse to fear. After swallowing our daily dose of London street dust, we need hardly hesitate to lick a stamp—even a green one.

Leeds is suffering from an outbreak of typhoid. It is understood that its origin has been traced to milk.

The Countess Boni de Castellane has erected a building, called the Galeries de la Charité, close to the Champs Elysées, to be devoted to charitable purposes, such as the holding of meetings, bazaars, and entertainments in aid of the institutions.

A cottage hospital has been opened at Aberlour, Banffshire, erected and endowed under the will of the late Mr. James Fleming, of the same place. The centre of the block of buildings which constitute the hospital is occupied by the administration. A corridor runs the length of the building. On each side are the wards, operating-room, duty-rooms, and usual offices.

The Bishop of Oxford has accepted the post of chaplain to the Radcliffe Infirmary, Oxford. At the meeting, at which the Bishop's acceptance was announced, one of the gentlemen present remarked that he understood that the chaplain to a hospital required the tact of a diplomatist and the temper of a saint. Under these circumstances it is a very fine act on the Bishop's part to undertake such an office.

The British residents in Pekin have opened a hospital there as a Jubilee Memorial. The institution is for the use of foreign patients, but there are also private wards, and it is in the hands of British trustees, and will be nursed by English nurses. A great deal of interest has been created locally, and many gifts in kind, as well as money subscriptions, have been made. A German firm furnished one of the private wards as an offering on their part.

The New Mater Infirmorum Hospital at Belfast is now open. It is constructed on the pavilion system, and consists of three blocks, the centre of which contains the administration, a corridor connecting it with the ward blocks on either side. Each pavilion consists of three storeys, and on each floor are two large wards. The pavilions are built on arches. The accommodation is for 150 beds and a certain number of cots. To each bed 1,300 cubic feet of air space is allowed. Sanitary towers and other modern arrangements have been provided. The roofs are of flat vulcanite, and laid out as gardens. Terrazzo is used in wards and passages.

The Willesden Parish Infirmary will be a very spacious institution. It is intended to house 616 patients. The administration block is, as usual, in the centre. From it runs a corridor, on each side of which the wards are thrown out. The whole is two storeys high. There are two small receiving wards and two small observation wards on each side of the administration block opening into the main corridors. Behind the main buildings there will be laundry, boiler-house, mortuary, lunatic ward, and lying-in ward, each in a separate building. It is intended eventually to add a nurses' home. The corridor walls will be of coloured glazed bricks; the floors tiled. The ward floors will be of hard white maple, and the walls plastered and painted. The lighting will be by electricity, the heating by means of open fires, supplemented by hot-water pipes, and the ventilation will be natural by means of windows, fan-lights, and flues.

